# PReS-FINAL-2129: Whole-body versus localized magnetic resonance imaging in the assessment of juvenile dermatomyositis

**DOI:** 10.1186/1546-0096-11-S2-P141

**Published:** 2013-12-05

**Authors:** A Madeo, MB Damasio, M Dellepiane, A Providenti, D Beleva, A Buoncompagni, A Beltramo, N Ruperto, A Martini, C Malattia

**Affiliations:** 1 Pediatrics, Istituto G. Giannini, Genova, Italy; 2 Radiology, Istituto G. Giannini, Genova, Italy

## Introduction

MRI represents a promising tool in the assessment of disease activity in juvenile dermatomyositis (JDM). Water-sensitive sequences are very sensitive to the presence of inflammation and have the potential to support diagnosis, guide muscle biopsy and monitor treatment response. So far, all MRI studies in JDM focused on pelvic and thigh musculature. Whole-body(WB)-MRI screens the entire body with the advantage to evaluate much larger areas of muscles as well as subcutaneous fat tissue.

## Objectives

To compare WB-MRI and thighs-MRI in the assessment of disease activity in JDM.

## Methods

WB-MR images were obtained from 43 JDM patients and 43 controls using a 1.5 Tesla MRI scanner and STIR sequences. Muscle signal abnormalities were scored by 2 independent readers in 36 muscular groups using a 0-2 point scale; perifascicular and subcutaneous tissue inflammation were evaluated using a binary scale on 8 sites (arm, forearm, thigh and lower leg bilaterally). Two different readers separately scored, on the same WB-MR images, pelvic and thigh muscles bilaterally (gluteal muscles, hamstrings, quadriceps and adductors) as well as the presence of thigh subcutaneous soft-tissue oedema and perifascicular oedema. WB-MRI and thighs MRI scores were compared in terms of reliability, construct validity, discriminant ability and responsiveness.

## Results

Thighs subcutaneous and myofascial signal abnormalities were detected in 8/43 (18.6%) and in 12/43 (27.9%) patients respectively. WB-MRI revealed inflammatory involvement of myofascial and subcutaneous areas other than the thigh in 8/43 (18.6%) and 10/43 (23.2%) patients, respectively Concordance between WB and thigh MRI scores in the evaluation of myofascial and subcutaneous tissue was moderate (myofascial scores r_s _= 0.59, subcutaneous scores r_s _= 0.69). Although concordance in detecting muscle inflammation between thigh and WB-MRI muscle scores was excellent (r_s _= 0.97), two patients with negative thigh-MRI showed muscle signal abnormalities in muscle groups different from the thigh. Inter-reader agreement was excellent for both thigh-MRI and WB-MRI scores (ICC 0.96 and 0.98 respectively). Both scores showed excellent correlations with clinical measures of disease activity (Manual Muscle Test (MMT) r_s _= -0.82, Childhood Myositis Assessment Scale (CMAS) r_s _= -0.83 for thigh-MRI; MMT r_s _= -0.84, CMAS r_s _= -0.81 for WB-MRI). Thigh and WB-muscle scores were significantly higher in JDM active patients when compared with the control group (pb < 0.0001 for both the scores) and the inactive patients (thigh-MRI pb = 0.0022, WB-MRI pb = 0.0037). Responsiveness to change was higher for WB-MRI muscle score (standardized response mean = 1.65) compared to that of thigh MRI score (SRM = 1.04) and to those of clinical muscle tests (SRM-CMAS = 0.56, SRM-MMT = 0.74).

## Conclusion

WB-MRI enables a reliable analysis of the site and magnitude of inflammatory process throughout the entire body thus providing a complete assessment of total inflammatory burden. WB-MRI was more accurate than localized MRI in identifying myofascial and subcutaneous inflammation and in the assessment of the responsiveness to change.

## Disclosure of interest

None declared.

